# Prognostic and Predictive Value of Immune-Related Gene Pair Signature in Primary Lower-Grade Glioma Patients

**DOI:** 10.3389/fonc.2021.665870

**Published:** 2021-05-27

**Authors:** Kunjian Lei, Jingying Li, Zewei Tu, Feng Liu, Minhua Ye, Miaojing Wu, Yue Zhu, Min Luo, Li Lin, Chuming Tao, Kai Huang, Xingen Zhu

**Affiliations:** ^1^ Department of Neurosurgery, The Second Affiliated Hospital of Nanchang University, Nanchang, China; ^2^ Department of Comprehensive Intensive Care Unit, The Second Affiliated Hospital of Nanchang University, Nanchang, China; ^3^ East China Institute of Digital Medical Engineering, Shangrao, China; ^4^ Institute of Neuroscience, Nanchang University, Nanchang, China; ^5^ Department of Medical Social Work, Second Affiliated Hospital of Nanchang University, Nanchang, China

**Keywords:** lower-grade glioma, immune-related gene pairs (IRGPs), overall survival (OS), prognostic signature, ssGSEA

## Abstract

Immune-related gene pairs (IRGPs) have been associated with prognosis in various cancer types, but few studies have examined their prognostic capabilities in glioma patients. Here, we gathered the gene expression and clinical profile data of primary lower-grade glioma (LGG) patients from The Cancer Genome Atlas (TCGA), the Chinese Glioma Genome Atlas (CGGA, containing CGGAseq1 and CGGAseq2), the Gene Expression Omnibus (GEO: GSE16011), and Rembrandt datasets. In the TCGA dataset, univariate Cox regression was performed to detect overall survival (OS)-related IRGs, Lasso regression, and multivariate Cox regression were used to screen robust prognosis-related IRGs, and 19 IRGs were selected for the construction of an IRGP prognostic signature. All patients were allotted to high- and low-risk subgroups based on the TCGA dataset median value risk score. Validation analysis indicated that the IRGP signature returned a stable prognostic value among all datasets. Univariate and multivariate Cox regression analyses indicated that the IRG -signature could efficiently predict the prognosis of primary LGG patients. The IRGP-signature-based nomogram model was built, revealing the reliable ability of the IRGP signature to predict clinical prognosis. The single-sample gene set enrichment analysis (ssGSEA) suggested that high-risk samples contained higher numbers of immune cells but featured lower tumor purity than low-risk samples. Finally, we verified the prognostic ability of the IRGP signature using experiments performed in LGG cells. These results indicated that the IRGP signature could be regarded as a stable prognostic assessment predictor for identifying high-risk primary LGG patients.

## Introduction

Gliomas are characterized by high recurrence rates and high mortality and are the most common primary intracranial tumors that occur in the adult central nervous system (1). Gliomas can be divided into 4 grades (Grades I–IV) according to the World Health Organization (WHO) classification system (2). Lower-grade gliomas (LGGs; WHO Grade II or III astrocytomas and oligodendrogliomas) are well-differentiated but may deteriorate to higher grades (Grade IV) over time (3, 4). In recent years, despite advances in diagnostic and therapeutic modalities for primary LGGs, patient prognosis has not improved (5). Therefore, the identification of new and significant targets or biomarkers to enhance the treatment effect and improve public awareness of LGGs management is urgent.

In recent studies, some elements of the immune system, such as the tumor microenvironment (TME), immune-related genes (IRGs) and immune cells have been found to play key roles in the formation and progression of tumors (6–8). The TME is highly associated with tumorigenesis, development, and prognosis, and immune cells play critical roles on tumor formation and progression (6, 9, 10). Recently, despite many challenges and doubts, many promising preclinical and clinical immunotherapy measures have been emerged for the treatment and management of glioma, including active and passive immunotherapy, gene therapy, and immune-checkpoint inhibitors, further revealing the important role played by immunotherapy in glioma treatment (11). Increasing evidence has indicated the significant contribution of IRGs to the complex regulatory network of cancer. IRGs have also been regarded as biomarkers to predict prognosis of cancer patients (12). A recent study constructed a stable IRG signature that was found to serve as an effective and predictive prognostic model among primary LGG patients (13).

In this study, we identified the prognostic significance of IRGPs in 476 primary LGG patients obtained from The Cancer Genome Atlas (TCGA) dataset. These findings have been confirmed in four external independent datasets (Chinese Glioma Genome Atlas [CGGA]: CGGAseq1 and CGGAseq2; Gene Expression Omnibus [GEO]: GSE16011, and Rembrandt). Furthermore, the results of gene set enrichment analysis (GSEA) and biological process analysis may provide additional clues regarding the potential functions of these IRGPs during glioma pathogenesis. In addition, we also built a nomogram model based on patient age, the risk score and WHO grade for the prediction of 1-, 3-, and 5-year overall survival (OS) rates among primary LGG patients. IRGPs were found to be strong prognostic biomarkers and predictors in primary LGG patients.

## Materials and Methods

### Data Acquisition

In our study, we collected five independent glioma cohorts, including the TCGA cohort, two CGGA cohorts (CGGAseq1 and CGGAseq2), the Rembrandt cohort, and the GSE16011 cohort. The RNA-seq data for the TCGA cohort was acquired from the Genomic Data Commons Data Portal website (GDC; https://portal.gdc.cancer.gov/), and the relevant clinical data were obtained from the cBioPortal website (https://www.cbioportal.org/).

The mRNA expression data and clinical information for the CGGAseq1 and CGGAseq2 cohorts were retrieved from the CGGA website (http://www.cgga.org.cn/). The microarray data for the GSE16011 and Rembrandt validation cohorts were obtained from the Gene Expression Omnibus (GEO) repository (https://www.ncbi.nlm.nih.gov/geo/), and relevant clinical data were acquired from a previous publication (14). The list of 2,497 IRGs was obtained from the MSigDB (https://www.gsea-msigdb.org/gsea/msigdb/index.jsp) (15) and ImmPort (https://www.immport.org/home) websites.

### Patient Exclusion Criterion

The criteria for including glioma patients were as follows: (a) primary glioma patients with OS > 1 month; (b) patients with expression data; and (c) patients with WHO Grade II or III gliomas. By applying these inclusion criteria, we obtained five independent cohorts (TCGA, CGGAseq1, CGGAseq2, GSE16011, and Rembrandt), which included 476, 270, 137, 102, and 129 primary LGG patients, respectively. The basic information for the selected patients is shown in **Table 1**.

**Table 1:** The basic information for primary low-grade glioma (LGG) patients in the TCGA, CGGAseq1, CGGAseq2, GSE16011, and Rembrandt datasets.

Variable	TCGA set (n=476)		CGGAseq1 set (n=270)		CGGAseq2 set (n=137)		GSE16011 set (n=102)		Rembrandt set (n=129)	
**Age**										
>=mid	245	51.47%	143	52.96%	72	52.55%	58	56.86%	67	51.94%
<mid	231	48.53%	127	47.04%	65	47.45%	54	52.94%	62	48.06%
**Gender**										
Male	260	54.62%	150	55.56%	85	62.04%	67	65.69%	77	59.69%
Female	216	45.38%	120	44.44%	52	37.96%	35	34.31%	52	40.31%
**Grade**										
WHO II	231	48.53%	130	48.15%	90	65.69%	22	21.57%	64	49.61%
WHO III	245	51.47%	140	51.85%	47	34.31%	80	78.43%	65	50.39%
**IDH1**										
Wildtype	85	17.86%	176	65.19%	35	25.55%	37	36.27%	N/A	N/A
Mutant	388	81.51%	64	23.70%	101	73.72%	44	43.14%	N/A	N/A
N/A	3	0.63%	30	11.11%	1	0.73%	21	20.59%	N/A	N/A
**1p/19q**										
Codel	157	32.98%	81	30.00%	50	36.50%	N/A	N/A	N/A	N/A
Non-codel	323	67.86%	157	58.15%	85	62.04%	N/A	N/A	N/A	N/A
N/A	0	0.00%	32	11.85%	2	1.46%	N/A	N/A	N/A	N/A

N/A, not applicable.

### Data Processing

The fragments per kilobase of transcript per million (FPKM) data from the three RNA-seq cohorts were transformed into transcripts per kilobase million (TPM) values using a previous publicly available algorithm (16, 17). These TPM values were then used in the later experiments. The undisposed data of these microarray cohorts were obtained to perform background adjustment and quantile normalization using a robust multiarray averaging method (RAM) with the “affy” (18) and “simpleaffy” (19) packages.

### Construction of the IRGP Signature

The training set obtained from TCGA was used to screen OS-related IRGs using univariate Cox regression analysis, and a total of 1,007 OS-related IRGs were identified (p < 0.001). Those IRGs with median absolute deviation (MAD) < 0.5 were excluded to calculate the IRGP index. A pairwise comparison translation was performed between OS-related IRG expression values to obtain an index for each IRGP in each sample. The IRGP was assigned a value of 1 if the expression of the former IRG was higher than that of the latter IRG; else, the index was defined as 0. IRGPs with gene ratios (1/0 or 0/1) > 0.4 and < 0.6 were retained. Univariate Cox regression was performed again to obtain OS-related IRGPs. Next, the Lasso Cox regression method, a recommended dimensionality-reduction method for the regression of high-dimensional data, was performed to select the OS-related IRGPs without multicollinearity. Finally, an IRGP-based risk signature was established by multivariate Cox regression, and an equation was produced to calculate risk scores for LGG patients with relevant IRGP indexes and respective coefficients. The following equation was obtained:

$$\mathrm{risk score}=\sum_{i=1}^{n}\mathrm{Coef}i*x_{i}$$

in which *Coef_i_* is the coefficient of each IRGP index, and *x_i_* is the IRGP index value for each screened IRGP among five cohorts.

### Functional Enrichment Analysis

Differentially expressed genes (DEGs) between the low- and high-risk primary LGG patients were identified using the “limma” package (20) and defined using the standards of |log_2_ (fold-change)| >1 and p < 0.05 in the TCGA cohort. A total of 3,673 genes were defined as DEGs and were used to perform gene-ontology biological-processes (GO-BP) and Kyoto Encyclopedia of Genes and Genomes (KEGG) analyses using by the “clusterProfiler” R package (21). The GSEA software (version 4.0.1, https://www.gsea-msigdb.org/gsea/index.jsp) (22) was used to perform GSEA to identify tumor hallmarks enriched in primary LGG patients with higher IRGP scores. Tumor hallmarks with |Normalized Enrichment Score (NES)| > 1.5, normalized p-value < 0.05, and false-discovery rate (FDR) q-value < 0.25 were defined as significantly enriching tumor hallmarks.

### Single-Sample Gene-Set Enrichment Analysis (ssGSEA)

A single-sample GSEA (ssGSEA) algorithm was used to quantify the infiltration of each immune cell type in the LGG TME using the R package “GSVA” (23). To identify TME infiltrating immune cells, gene sets from previous studies were downloaded (24, 25). The values acquired by ssGSEA represent the relevant abundance of each infiltrating immune cell in each sample.

### Construction and Validation of the Nomogram Model

The establishment and validation of a nomogram model were accomplished using the package “rsm.” The continuous variables of risk score, patient age and WHO grade, based on the findings of the multivariate Cox regression, were contained in our nomogram model. The “calibrate” function of the “rms” package was performed to generate calibration plots.

### Cell Culture

SW-1088, SW-1783, and Bt142-mut human glioma cell lines were purchased from American Type Culture Collection (ATCC). Normal human astrocytes (NHA) were acquired from the Culture Collection of the Chinese Academy of Sciences (Shanghai, China). SW-1088 and SW-1783 was cultured in Leibovitz’s L-15 Medium (Gibco), BT142-mut was cultured in DMEM/F12 with an additional 0.9% glucose, 4 mM L-glutamine (ATCC), 25 µg/mL insulin, 100 µg/mL transferrin, 20 nM progesterone, 15 µM putrescine and 30 nM selenite, all of these Medium were supplement with 10% fetal bovine serum (FBS; Gibco). All cells were maintained at 37°C in an incubator containing 5% CO_2_.

### Western Blotting Analysis

The cells were harvested and lysed with radioimmunoprecipitation assay (RIPA) cell lysis buffer. 6%–12% sodium dodecyl sulfate-polyacrylamide gel electrophoresis (SDS-PAGE) and polyvinylidene difluoride (PVDF) membranes were used to separate and transfer protein lysates (25 ng). The membranes were incubated with primary antibodies, including endothelial growth factor receptor (EGFR, 1:10,000, Proteintech), slit guidance ligand 1 (SLIT1, 1:1,000, Abcam), beta-2 adrenergic receptor (ADRB2, 1:1,000, CST), macrophage scavenger receptor 1 (MSR1, 1:1,000, CST), and glyceraldehyde 3-phosphate dehydrogenase (GAPDH, 1:2,000, Proteintech). The membranes were then incubated with horseradish peroxidase (HRP)-conjugated anti-rabbit and anti-mouse secondary antibodies corresponding to the primary antibodies. Finally, the bands were visualized with the enhanced chemiluminescence (ECL) substrate (Thermo) by GV6000M (GelView 6000pro). The intensities of the protein bands Were quantified (ImageJ software) and standardized against the levels of GAPDH.

### Quantitative Real-Time PCR (qRT-PCR)

TRIzol reagent (Thermo) was used to extract total RNA from cells. RNA was reverse transcribed into cDNA using High Capacity cDNA Reverse Transcription Kits (Bio-Rad). Quantitative real-time polymerase chain reaction (qRT-PCR) was performed using the following primer sequences: EGFR: F primer, 5’-AGGCACGAGTAACAAGCTCAC-3’, R primer, 5’-ATGAGGACATAACCAGCCACC -3’; SLIT1: F primer, 5’-GCCTGGAACTCAATGGCAAC-3’, R primer, 5’-CTGGTTTCGGTTCAGTCGCA-3’; ADRB2: F primer, 5’-TTGCTGGCACCCAATAGAAG-3’, R primer, 5’-CAGACGCTCGAACTTGGCA-3’; MSR1: F primer, 5’-GCAGTGGGATCACTTTCACAA-3’, R primer, 5’-AGCTGTCATTGAGCGAGCATC-3’; and GADPH: F primer, 5’-CTCACCGGATGCACCAATGTT-3’, R primer: 5’-CGCGTTGCTCACAATGTTCAT-3’. The relative mRNA expression levels were analyzed using GraphPad Prism 8 software after normalizing against the expression level of GAPDH.

### Statistical Analysis

We implemented Kaplan-Meier method to contrast the survival outcomes between low and high IRGP score LGG patients by using the two-sided log-rank statistical test. Receiver operating characteristic (ROC) model (time-dependent) was utilized to evaluate the prognostic predictive power of the IRGP score. The prognostic value of IRGPs were assessed by univariate Cox regression analysis. The independent prognostic role of IRGP-signature was determined by univariate and multivariate Cox regression in each cohort. Statistical analyses were performed by the R program langage (version 3.6.1, https://www.r-project.org/) and SPSS Statistics software (version 25, https://www.ibm.com/products/software) in this study.

## Results

### Identification and Construction of IRGP Index in Primary LGG Patients

**Figure 1:**
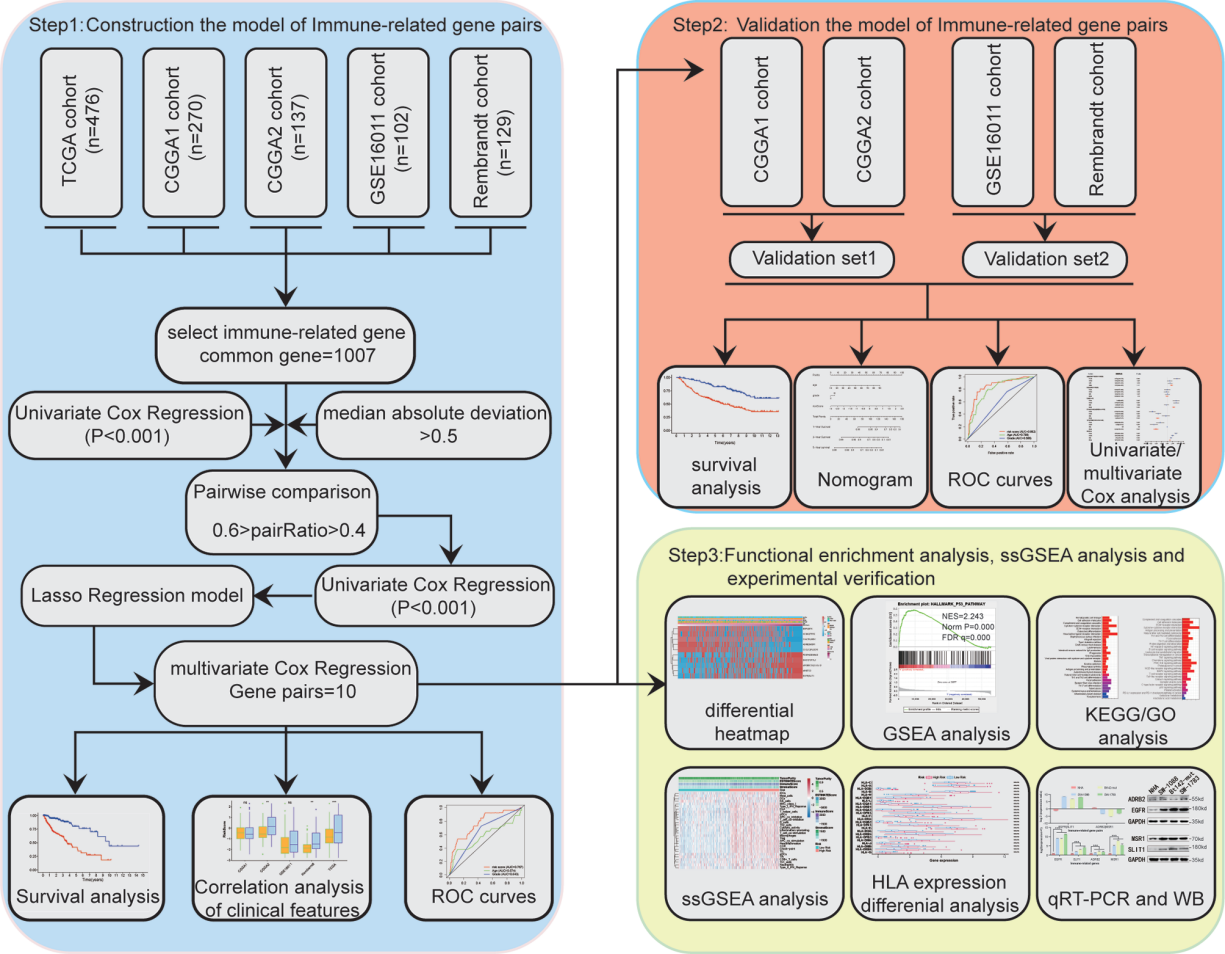
Study flow chart.

**Figure 2:**
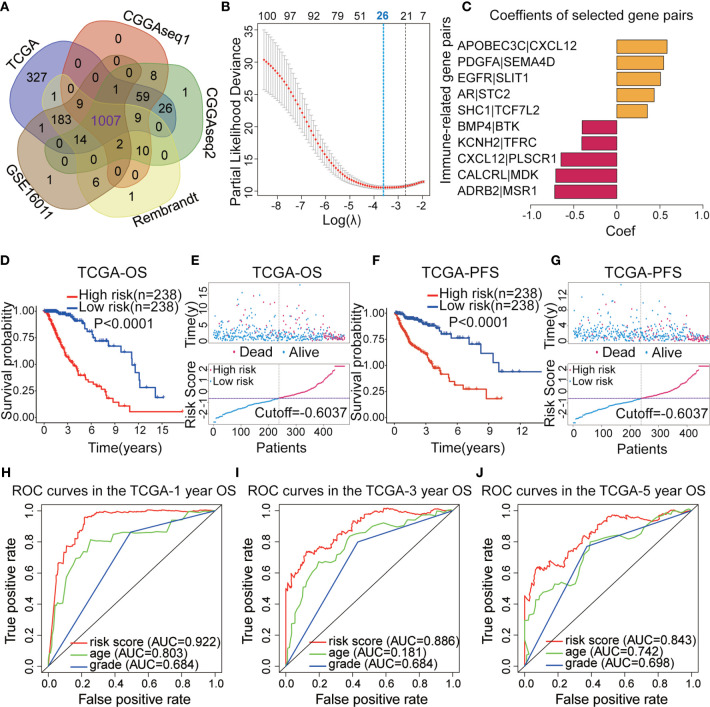
**(A)** Venn diagram showing the 1,007 immune-related genes (IRGs) screened in all five datasets. **(B)** Least absolute shrinkage and selection operator (Lasso) regression were performed to calculate the minimum criteria. **(C)** Multivariate Cox analysis was used to calculate the coefficients of the IRG pairs (IRGPs). **(D)** Kaplan–Meier (KM) survival curves revealed that the low-risk subgroup had better overall survival (OS) than the high-risk subgroup in The Cancer Genome Atlas (TCGA) dataset. **(E)** Survival status and distributions of risk scores (based on the IRGP signature) of primary low-grade glioma (LGG) patients in TCGA dataset. **(F)** KM curves showed that the high-risk subgroup had a worse platinum-free interval (PFI) than the low-risk subgroup in the TCGA dataset. **(G)** Survival status and risk score distribution scatter plots for primary LGG patients in TCGA dataset. **(H–J)** Receiver operating characteristic (ROC) curves for the IRGP signature for the prediction of 1/3/5-year survival in the TGGA dataset.

**Figure 1** displays the workflow used for our study process. First, the IRG expression matrix were screened from all five datasets, and then the intersecting IRGs among all datasets were identified using a Venn diagram. A total of 1,107 IRGs were detected in all five datasets (**Figure 2A**). For a better analysis, the TCGA dataset was served as our training set and applied a univariate Cox regression analysis (p < 0.001) to screen the identified IRGPs in the TCGA dataset, resulting in the identification of 327 IRGs. After determining the value of IRGPs, which were calculated by these screened IRGs, we obtained a final set of 1,124 OS-related IRGPs. In order to build the relationship between OS outcomes and IRGPs, univariate Cox regression analysis was performed and recognized 101 IRGPs that were noticeably correlated with patient prognosis. To identify robust IRGPs, we used Lasso Cox regression analysis and multivariate Cox regression analysis on the training set. Finally, we extracted 10 IRGPs (**Figures 2B, C**) and 19 IRGs, including 5 negative and 5 positive gene pairs. The Cox regression analysis results were used to confirm the IRGP signature for predicting the OS among primary LGG patients. The coefficients and univariate Cox analysis results for these 10 IRGPs are shown in **Table S1**. Risk score was calculated for each patient on the basis of the developed risk model. We selected an optimal cut-off value of 0.6037 for the IRGP index to predict primary LGGs prognosis by using time-dependent ROC curve analysis. Using the determined cut-off value as the threshold, patients were divided into low- and high-risk subgroups in the TCGA dataset, and the Kaplan-Meier survival curves showed that primary LGG patients with lower risk scores had better clinical outcomes (higher OS rates and a longer OS times, **Figure 2D**, p < 0.001). The survival status and risk scores distributions are shown in **Figure 2E**. In addition, we analyzed the progression free survival (PFS)of the patients, which returned a similar conclusion (**Figure 2F**, p < 0.001). The survival status and risk score distributions are shown in **Figure 2G**.

To confirm the predictive ability of the IRGP signature, we used the risk scores from the TCGA dataset to build a The ROC curves indicated that IRGPs might provide the reliable power for the prediction of OS among the training dataset (1-year area under the ROC curve [AUC] = 0.922, 3-year AUC = 0.886, 5-year AUC = 0.843; **Figures 2H–J**).

### Validation of the Prognostic Value of IRGP-Signature

**Figure 3:**
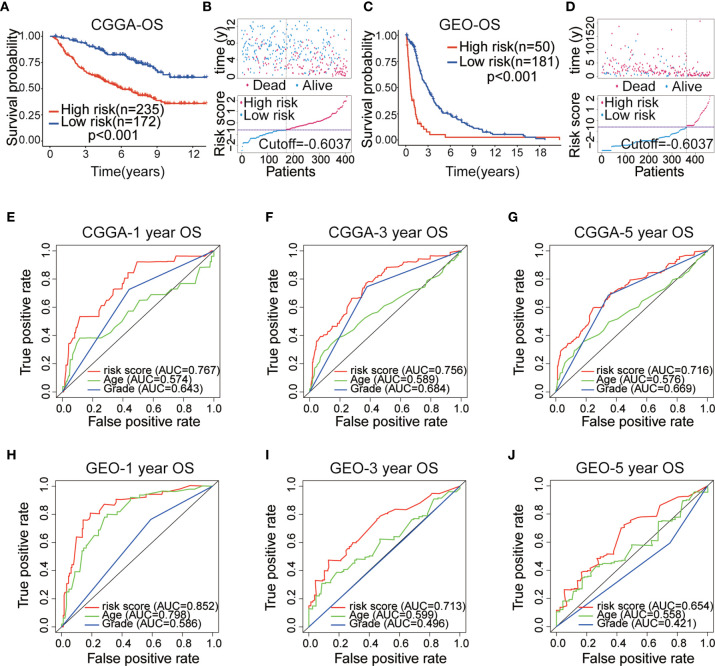
**(A)** Kaplan–Meier (KM) survival curves of 10 immune-related gene pairs (IRGPs) in the China Glioma Genome Atlas (CGGA) validation dataset. **(B)** Survival status and risk score distribution scatter plots of primary low-grade glioma (LGG) patients in the CCGA dataset. **(C)** KM survival curves of 10 IRGPs in the Gene Expression Omnibus (GEO) validation dataset. **(D)** Survival status and risk score distribution scatter plots of primary LGG patients in the GEO dataset. **(E–G)** ROC curves of the IRGP signature for predicting 1/3/5-year survival in the CGGA dataset. **(H–J)** ROC curves of the IRGP signature for predicting 1/3/5-year survival in the GEO dataset.

After establishing the prognostic model, we merged the other four datasets into two external validation sets (CGGAseq1 and CGGAseq2 were combined into the CGGA dataset, whereas the GES16011 and Rembrandt datasets were combined into the GEO dataset). Risk scores were calculated for patients in the GEO and CGGA datasets using the formula applied to the TCGA dataset to validate the prognostic ability of the IRGP signature. And primary LGG patients were divided into high- and low-risk subgroups in the CGGA and GEO datasets based on the training set’s median value risk score. As shown in **Figures 3A**, **C** (p < 0.001), the results were consistent for these independent data sets were consistent with the findings from the training dataset. Primary LGG patients in the low-risk subgroup had higher survival rates and longer OS times in both of the validation datasets. Survival status and risk score distributions are shown in **Figures 3B**, **D**, indicating that higher mortality and shorter overall survival times will be seen among patients with higher risk scores. In order to better investigate the effect of treatment on the prognosis of LGG patients, we added the influence of clinical treatment on patients between high-risk group and low-risk group based on IRGPs signature in the CGGA dataset. And then we further divided two groups into three subgroups: 1. No special treatment; 2. Radiotherapy or chemotherapy; 3. Radiotherapy and chemotherapy. The results showed that there was no significant difference among the three treatments in the low-risk group, but there was significant difference in the high-risk group (**Figures S1A, B**). This finding implies that our model can guide clinical treatment and predict prognosis to a certain extent.

The ROC analysis was used to assess the accuracy of 10 IRGP-based risk score prediction in the GEO and CGGA datasets. The results displayed that the IRGP signature was a reliable predictor in the CGGA dataset (1-, 3-, 5-year AUC = 0.767, 0.756 and 0.716, respectively; **Figures 3E–G**) and GEO dataset (1-, 3-, 5-year AUC = 0.852, 0.713 and 0.654, respectively; **Figures 3H–J**). These results demonstrated that the IRGP signature presented a robust prognostic value for multiple cohorts of primary LGG patients.

### Predictive Abilities of Risk Models for Different Clinical Sample Characteristics

**Figure 4:**
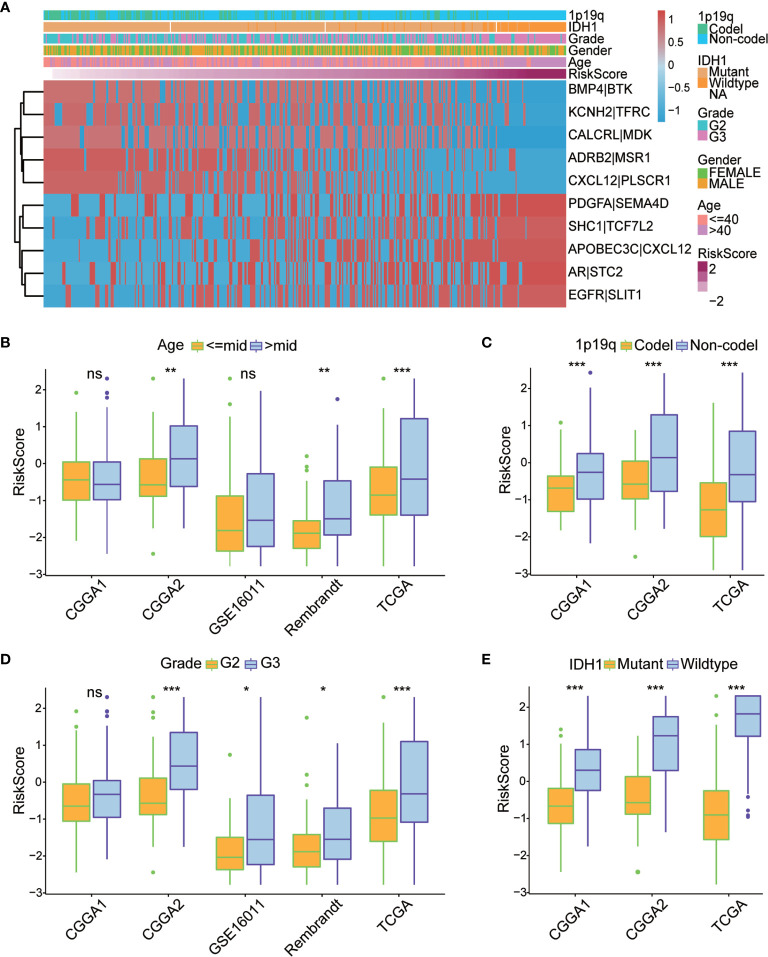
**(A)** Heatmap of the correlation between the 10 immune-related gene pairs (IRGPs) and the clinicopathological features of The Cancer Genome Atlas (TCGA) dataset. **(B–E)** Box plots revealed that patients with different clinicopathological features (including age, 1p/19q co-deletion status, WHO grade, and IDH mutation status) had different risk scores between the high- and low-risk subgroups. World Health Organization; IDH, isocitrate dehydrogenase. *P < 0.05, **P < 0.01, ***P < 0.001, ns, no significant difference.

To evaluate the robustness of each of the 10 IRGPs for predicting various clinical characteristics in primary LGG patients, a heatmap was designed to visualize the correlations between the identified IRGPs and common clinical features of LGGs, including age, sex, WHO grade, IDH mutation status, 1p/19q co-deletion status, and risk score (**Figure 4A**). We found that gene expression ratios of 1 for ADRB2|MSR1, CALCRL|MDK, CXCL12|PLSCR1, KCNH2|TFRC, and BMP4|BTK were negatively correlated with high risk in primary LGG patients, whereas gene expression ratios of 1 for SHC1|TCF7L2, AR|STC2, EGFR|SLIT1, PDGFA|SEMA4D, and APOBEC3C|CXCL12 were positively correlated with high risk among primary LGG patients, suggesting that these IRGPs may be potentially detrimental in primary LGG patients who express them. Meanwhile, we found that some clinical characteristics, including age > 40 years, 1p/19q non-codeletion, IDH wild-type, and WHO grade III, were enriched in the high-risk subgroup; however, no difference in gender was identified between two subgroups. Box plots were used to visualize the relationships between risk score and each of the examined clinical characteristics among all five datasets (**Figures 4B–E**, **Figure S1I**). In order to assess the prognostic roles of these different clinical characteristics in primary LGG patients,

### Independent Prognostic Value of the IRGP Signature Stratification

**Figure 5:**
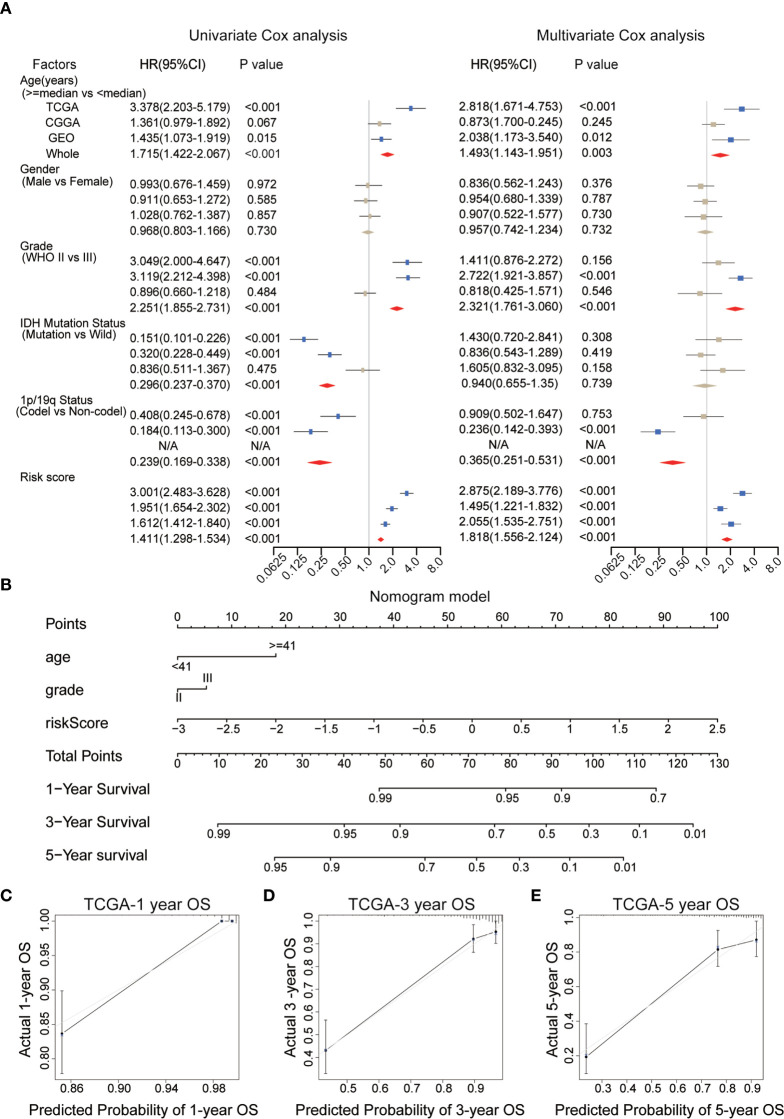
**(A)** Univariate and multivariate Cox analyses of clinicopathological and molecular features, including age, gender, WHO grade, IDH mutational status, 1p/19q co-deletion status, and risk scores, in each dataset. **(B)** A nomogram was established to predict the overall survival (OS) of primary low-grade glioma (LGG) patients at 1/3/5 years. **(C–E)** Calibration curves for the nomogram for predicting the OS probability at 1/3/5 years among primary LGG patients in the TCGA dataset. WHO, World Health Organization; IDH, isocitrate dehydrogenase; TCGA, The Cancer Genome Atlas.

To evaluate the independent prognostic value of the IRGP signature for the TCGA dataset and the four validation datasets (CGGAseq1, CGGAseq2, GSE16011, and Rembrandt), univariate and multivariate COX regression analyses were performed and we systematically analyzed their clinical information, including age, sex, WHO grade, IDH mutation status, 1p/19q co-deletion status, and the risk score based on the 10 IRGPs to determine hazard ratios (HR), 95% confidence intervals (Cis), and p-values (**Figure 5A**).

We found that the risk score, age, IDH mutation status, WHO grade and 1p/19q co-deletion status were correlated with survival in the TCGA dataset, and most of these results could be reproduced in the other datasets. Risk score, age and WHO grade were positively correlated with final risk, suggesting the growth of risk prediction, in contrast with the 1p/19q co-deletion status and IDH mutation status. However, only the risk score (HR = 1.818, 95% CI: 1.556–1.214, p < 0.001), but no other clinical information, was significantly and consistently associated with survival across all tested datasets. These results powerfully suggested that the IRGP signature can be explored for use in clinical applications to predict the prognosis of primary LGG patients as a potential OS predictor.

### Construction and Validation of a Nomogram

The WHO glioma grade, without doubt, has been correlated with patient prognosis, although multivariate regression analysis revealed no significant differences between two subgroups in the WHO grade of gliomas in the present analysis. Therefore, in order to evaluate the prognostic abilities of the IRGP signature, we selected clinical variables, including age, WHO grade, and the risk score for use as independent prognostic factors during the establishment of a nomogram model based on the training set (**Figure 5B**). In **Figure 5B**, longer lines represent the stronger effects of various influential factors. The risk score was associated with the longest line, indicating that the risk score had the most stable effect on the prediction of survival rate compared with the other two clinical characteristics. The calibration curves generated for this nomogram to forecast 1-, 3-, and 5-year survival rates had significant accuracy of prediction (**Figures 5C–E**). We found that OS prediction using the nomogram had stable predictive accuracy in the validation datasets (**Figures S1C–I**). In addition, The C-index of our nomogram model was 0.878 for the TCGA dataset, and we also obtained great C-index values for the CGGA (C-Index: 0.734) and GEO validation datasets (C-Index: 0.748). These findings suggest that the nomogram model based on IRGP signature had the dependable ability to act as a model for predicting outcomes of patients.

### Biological Processes Correlated With the IRGP Signature

**Figure 6:**
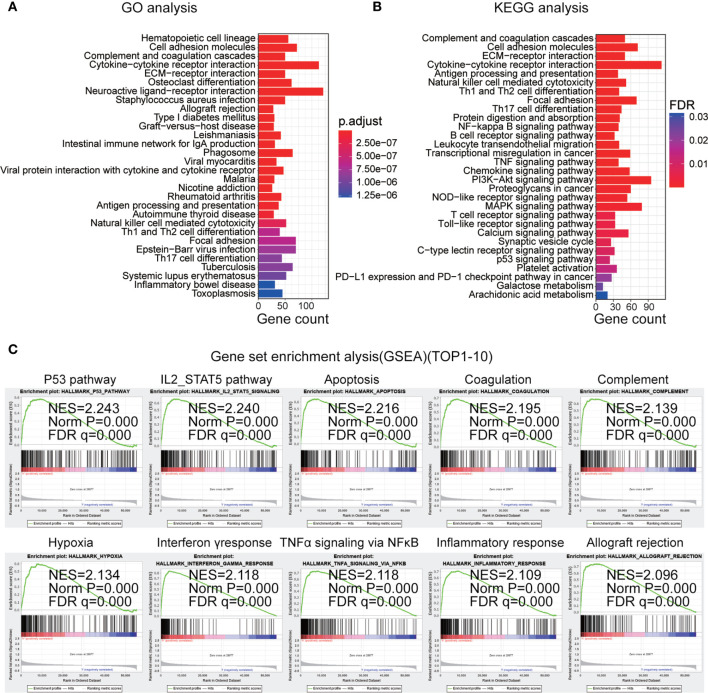
**(A)** Top 30 gene ontology biological process (GO-BP) analysis enrichment results based on differentially expressed genes. **(B)** Top 30 Kyoto Encyclopedia of Genes and Genomes (KEGG) pathway analysis enrichment results. **(C)** Top 10 gene set enrichment analysis (GSEA) results in the high-risk subgroup.

To study the underlying biological functions of the IRGP signature, differential expression analysis was used to identify DEGs between the high- and low-risk primary LGG patients in the training dataset. We selected DEGs (p < 0.05, |log_2_ (fold-change)| > 1) and used the R package “clusterProfiler” to perform KEGG and GO enrichment analysis based on the identified DEGs. We found these pathways were primarily enriched in immune-related biological processes, including cell adhesion molecules, T cell receptor signaling pathways, natural killer cell-mediated cytotoxicity and B cell receptor signaling pathways, and cancer-associated pathways, such as the phosphoinositide 3-kinase (PI3K)/protein kinase B (Akt) signaling pathway, the p53 signaling pathway, and the nuclear factor (NF)-κB signaling pathway. The top 30 most significantly enriched pathways are shown in **Figures 6A**, **B**. In addition, we performed GSEA between high- and low-risk primary LGG patients. The results identified 48 cancer hallmark gene sets in the high-risk subgroup, such as P53_PATHWAY, IL2_STAT5_SIGNALING, PI3K_AKT_MTOR_SIGNALING, and MTORC1_SIGNALING (**Figures 6C**, **S2A**). These enrichment analyses revealed that the IRGP signature might play a prognostic role in primary LGG.

### Single-Sample Gene Set Enrichment Analysis (ssGSEA)

**Figure 7:**
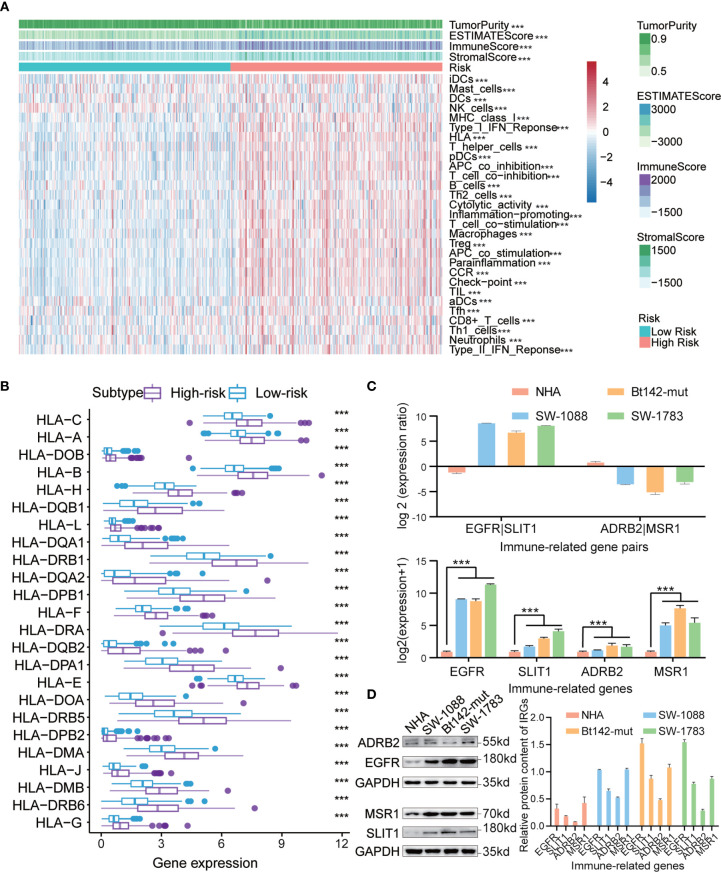
**(A)** Hierarchical clustering of primary low-grade glioma (LGG) patients into high- and low-risk subgroups based on the immune-related gene pair (IRGP) signature. Tumor purity, estimated scores, immune scores, and stromal scores were assessed by ESTIMATE, based on 29 immune-associated gene sets. **(B)** Comparison of human leukocyte antigen (HLA) genes between high- and low-risk subgroups. **(C)** qRT-PCR analysis of four IRGs in NHA, SW-1088, Bt142-mut, and SW-1783 cells. Expression was normalized against glyceraldehyde 3-phosphate dehydrogenase (GAPDH) mRNA expression. Data are presented as the mean ± SEM. **(D)** Western blotting analysis was performed to measure IRG protein levels in NHA, SW-1088, Bt142-mut, and SW-1783 cells. ***P < 0.001.

Based on the risk scores determined for the IRGP signature in the training dataset, all samples were divided into low- and high-risk subgroups, and unsupervised clustering analysis of 29 IRG sets was performed, with each sample obtaining an ssGSEA score. As shown in **Figure 7A**, the high-risk subgroup showed higher levels of IRGs than the low-risk subgroup. The stromal scores, immune scores, ESTIMATE (estimation of stromal and immune cells in malignant tumor tissues using expression data) scores, and tumor purity were significantly different (p < 0.0001) between two subgroups. We found that the tumor purity was markedly lower in the high-risk subgroup than in the low-risk subgroup. However, in the high-risk subgroup, the immune scores, ESTIMATE score and stromal scores were observably higher, which demonstrated opposite trends compared with tumor purity. These findings showed that low-risk samples contained lower numbers of immune cells and stromal cells but higher numbers of tumor cells than high-risk samples. In addition, we also analyzed the expression of human leukocyte antigen (HLA) in each subgroup. Interestingly, all HLA genes were expressed at higher levels in the high-risk subgroup (P < 0.0001, **Figure 7B**).

### Validation of the Transcription and Protein Expression Levels of IRGs

To further ascertain the believability of the IRGP signature in normal human astrocytes and low-grade glioma cells (WHO Grade III, astrocytoma), we performed western blotting analysis and qRT-PCR to investigate the transcription and protein expression levels of four selected IRGs (EGFR, SLIT1, ADRB2, and MSR1) in these cell lines. We discovered that these genes’ transcription and protein expression levels in normal human astrocytes were lower than those in LGG cells. In addition, we found that EGFR had higher transcription and protein expression levels compared with those for SLIT1 in gliomas characterized as WHO III in patients >40 years. However, an adverse trend was detected for the levels of ADRB2 and MSR1 (**Figures 7C, D**). The results observed for WHO Grade III I patients >40 years were consistent with the IRGPs we have obtained previously in high-risk patients (EGFR|SLIT1 = 1, ADRB2|MSR1 = 0).

## Discussion

In clinical settings, patients remain at risk for recurrence and death, even after sufficient surgical treatment, due to the malignant characteristics of glioma. Although temozolomide and other chemotherapy drugs have been shown to be effective for patient treatments, current protocols have difficulty predicting prognosis and targeting treatments during the early stages of glioma (26, 27). Therefore, reliable clinical biomarkers capable of accurately identifying patients and predicting prognosis remain necessary and practical to facilitate the better treatment of patients. In our study, we constructed an IRGP signature based on IRGs for predicting the outcomes of primary LGG patients and obtained significance in most analyses.

Much recent studies have focused on the relationships between IRG expression and the occurrence and development of various tumors (28). Deepening research has shown that IRGs have the stable ability to predict patient prognosis, and several IRGs with robust predictive functions have been reported (12). Interestingly, increasing attention has been given in checkpoint-inhibitor immunotherapy on account of its favorable effects for the management of solid tumors (29–32) and is expected to become a new option for LGG patients. To date, some existing models have used IRGs as a prognostic predictor of glioma patients (33, 34). A study examining the gene expression pattern of tumor immune infiltration identified an association with LGG malignancy, and a risk model based on 20 IRGs with different expression levels was shown to present decent OS prediction abilities for LGG (13). However, many prognosis-related models are limited by factors such as small sample size and the lack of sufficient validation.

Therefore, we selected five datasets (TCGA, CGGAseq1, CGGAseq2, GSE16011, and Rembrandt) from TCGA, CGGA, and GEO databases, resulting in the inclusion of 1,114 patients with primary LGG and the identification of 1,007 common IRGs with differential expression among all five datasets. After a series of analyses based on the TCGA dataset, 10 IRGPs were defined using 19 selected IRGs to build a prognostic model for primary LGG patients, and a risk score was calculated for each patient. According to the median value of the risk scores, patients were divided into low- and high-risk subgroups, and survival analysis revealed a noteworthy difference between two subgroups, with worse clinical outcomes for the high-risk group. In the correlation analyses with clinical characteristics, we detected distinct differences in the age, WHO grade, 1p/19q co-deletion status and IDH mutation status between the high- and low-risk subgroups. However, multivariate Cox regression revealed that the risk score constructed using the 10 IRGP signature was a stable, independent prognostic factor of LGG outcomes. In addition, we established nomograms, based on the IRGP signature risk score, age and WHO grade, for primary LGG patients in the training dataset. During the validation process, we found that the nomograms had robust abilities to predict the OS of patients in GEO and CGGA databases; although the prediction ability was slightly lower for the GEO dataset, the analysis revealed good performance. To further explore the potential biologic processes associated with the IRGP signature, we screened DEGs between the high- and low-risk subgroups and conducted GO, KEGG, and GSEA analyses. The results including cell invasion, proliferation, and immune and inflammatory responses, with many enriched pathways correlated with immune and inflammatory responses. Finally, based on the previously identified high-risk groups and 29 IRG sets, we used ssGSEA to examine the differential expression of HLA associated genes in primary LGG patients in the TCGA dataset. Not surprisingly, the results displayed that the high-risk subgroup was associated with increased immune cell infiltration and IRG expression than the low-risk subgroup, further confirming the key role played by immunity in the prognosis of primary LGG patients.

In this study, we selected five diverse datasets containing primary LGG patients, providing some advantages for the analysis methods and conclusions; however, some limitations remain. First, some clinical molecular features, such as O6-methylguanine (O6-MeG)-DNA methyltransferase (MGMT) status, which has been regarded as a robust predictor of prognosis in patients with glioma (35–37), were not included in the current analysis. In addition, the two GEO datasets (GSE16011 and Rembrandt) could not be further analyzed in our study due to the omission of information, including IDH mutation status and 1p/19q co-deletion status. In addition, our data were all obtained from open-access datasets. Although all data were analyzed after normalization to values of [0,1], due to differences between microarray and sequencing technology, some systematic errors likely remained. Moreover, our results were only studied and analyzed in retrospective datasets, with no analysis of a prospective dataset. Additionally, the ethnic groups included in the five datasets were not uniform, although the impacts of ethnic differences were minimized by the risk model during the validation process. The conclusions obtained from a series of limited bioinformatics analyses are insufficient and require further verification through comprehensive experiments and clinical studies. Furthermore, the functional and expression data of these IRGs in primary LGG patients remains necessary, preferably in larger and multiple datasets combined with additional clinical features.

In conclusion, our study constructed and comprehensively analyzed an IRGPs-based risk model. Combined with independent clinical factors, we established nomograms for predicting the prognosis of primary LGG patients. Furthermore, ssGSEA and HLA gene expression analyses revealed differences in immune cell infiltration and IRG expression between different risk subgroups. These findings indicate that this may be a valid method for assessing the prognostic risk of primary LGG patients and may represent a promising avenue to develop more effective treatments and management strategies for primary LGG patients.

## Data Availability Statement

The original contributions presented in the study are included in the article/**Supplementary Material**. Further inquiries can be directed to the corresponding authors.

## Author Contributions

XZ and KH constructed this study. KL, ZT, and JL performed the data analysis, figures plotted and writing. ML and LL did the western blotting and polymerase chain reaction experiments. FL, MY, YZ, and MW were responsible for the data acquisition and critical reading of the manuscript. All authors contributed to the article and approved the submitted version.

## Funding

The current study was funded by the National Natural Science Foundation (grant nos. 81960456, 82002660, 81760445 and 81760446); Jiangxi Province Department of Education Science and technology research project, China (grant no. GJJ190018).

## Conflict of Interest

The authors declare that the research was conducted in the absence of any commercial or financial relationships that could be construed as a potential conflict of interest.

## Supplementary Material

Supplementary Table 1 The coefficients and univariate Cox analysis results for 10 IRGPs in the TCGA, CGGA, and GEO datasets. *Red-color indicates risky IRGPs, whereas blue indicates protective IRGPs.*

Supplementary Figure 1
**(A, B)** Kaplan–Meier (KM) survival curves of three subgroups between high- and low-risk groups based on IRGPs model. **(C–E)** Calibration curves of the nomogram for predicting the OS probability at 1/3/5 years among primary low-grade glioma (LGG) patients in the China Glioma Genome Atlas (CGGA) dataset. **(F, G)** Calibration curves of the nomogram for predicting the OS probability at 1/3/5 years of primary LGG patients in the Gene Expression Omnibus (GEO) dataset. **(H)** Box plots revealed different risk scores between high- and low-risk subgroups of different sexes.

Supplementary Figure 2 Top 11-20 gene set enrichment analysis (GSEA) results in the high-risk subgroup.
