# Supplementary figures and images for: Prognostic and Predictive Value of Immune-Related Gene Pair Signature in Primary Lower-Grade Glioma Patients
Source: Front Oncol. 2021 May 27;11:665870. doi: 10.3389/fonc.2021.665870 (PMC8190397; doi:10.3389/fonc.2021.665870)

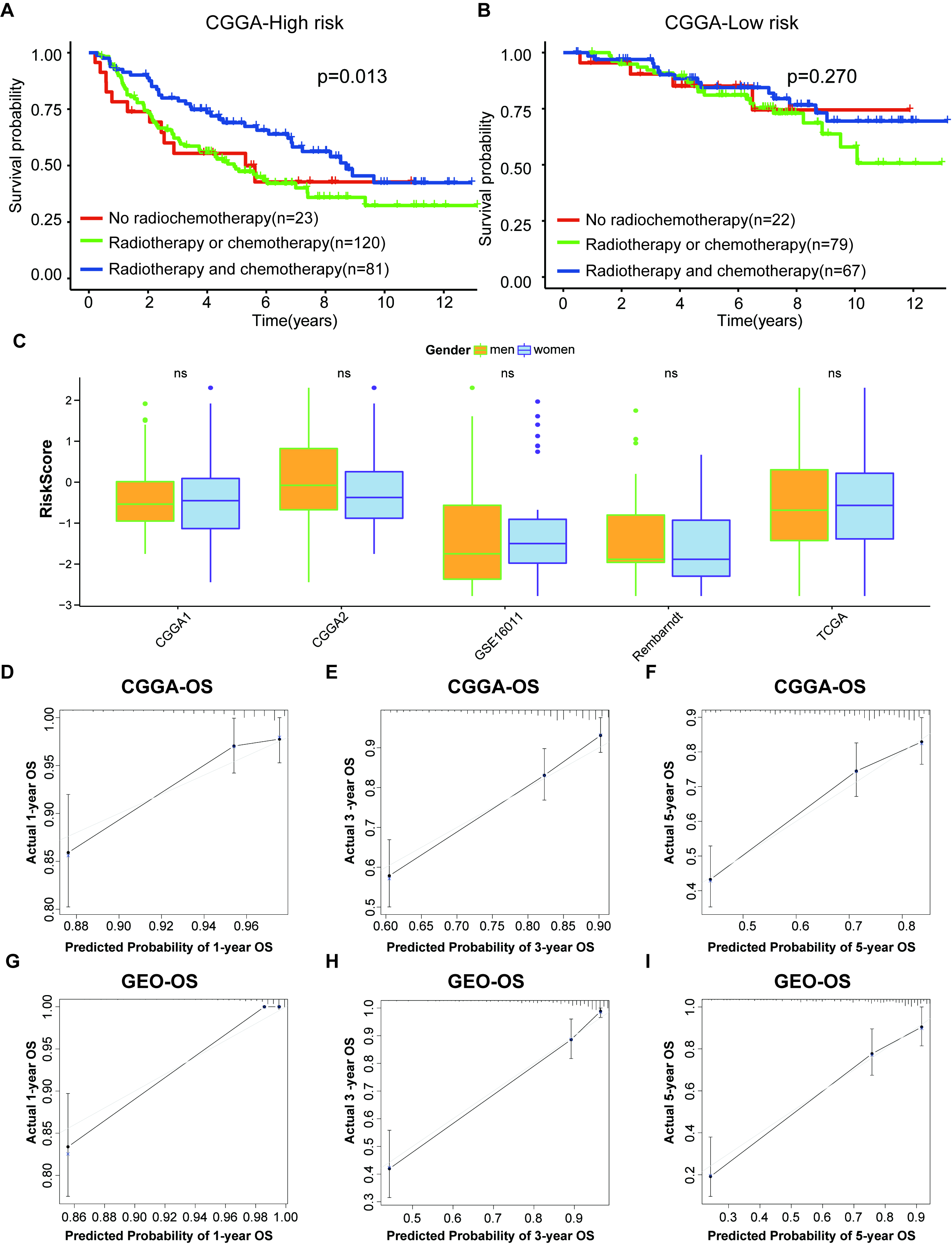

Supplement: Supplementary Figure 1 — (A, B) Kaplan–Meier (KM) survival curves of three subgroups between high- and low-risk groups based on IRGPs model. (C–E) Calibration curves of the nomogram for predicting the OS probability at 1/3/5 years among primary low-grade glioma (LGG) patients in the China Glioma Genome Atlas (CGGA) dataset. (F, G) Calibration curves of the nomogram for predicting the OS probability at 1/3/5 years of primary LGG patients in the Gene Expression Omnibus (GEO) dataset. (H) Box plots revealed different risk scores between high- and low-risk subgroups of different sexes. [file Image_1.tif]

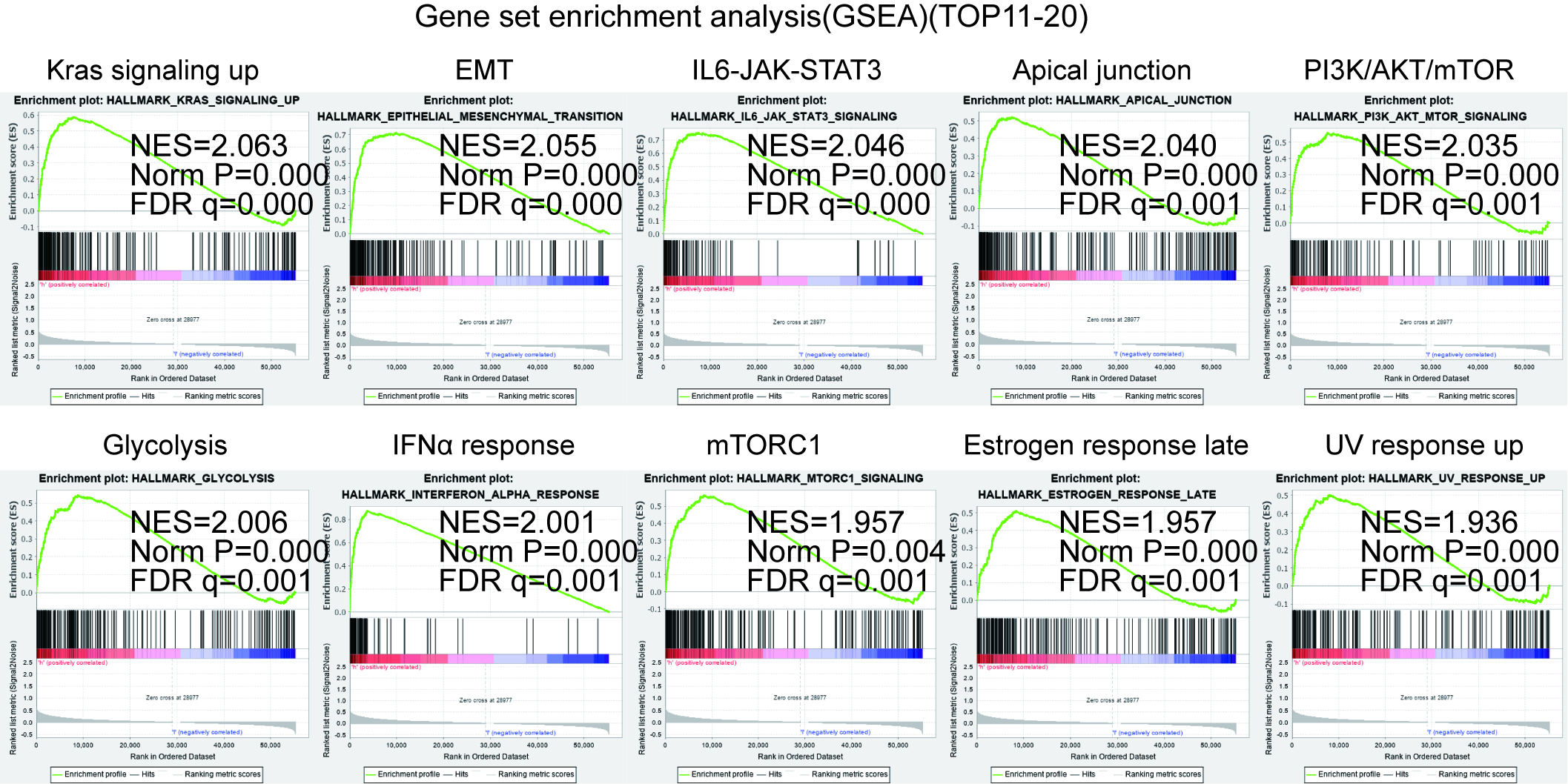

Supplement: Supplementary Figure 2 — Top 11-20 gene set enrichment analysis (GSEA) results in the high-risk subgroup. [file Image_2.tif]
